# Prognostic Nomogram for Liver Metastatic Colon Cancer Based on Histological Type, Tumor Differentiation, and Tumor Deposit: A TRIPOD Compliant Large-Scale Survival Study

**DOI:** 10.3389/fonc.2021.604882

**Published:** 2021-10-12

**Authors:** Le Kuai, Ying Zhang, Ying Luo, Wei Li, Xiao-dong Li, Hui-ping Zhang, Tai-yi Liu, Shuang-yi Yin, Bin Li

**Affiliations:** ^1^ Department of Dermatology, Yueyang Hospital of Integrated Traditional Chinese and Western Medicine, Shanghai University of Traditional Chinese Medicine, Shanghai, China; ^2^ Institute of Dermatology, Shanghai Academy of Traditional Chinese Medicine, Shanghai, China; ^3^ Center for Translational Medicine, Huaihe Hospital of Henan University, Kaifeng, China; ^4^ Department of Urology Surgery, Huaihe Hospital of Henan University, Kaifeng, China; ^5^ Institute of Evidence-Based Medicine and Knowledge Translation, Henan University, Kaifeng, China; ^6^ Research and Development Center, Shanghai Applied Protein Technology Co., Ltd., Shanghai, China; ^7^ Shanghai Skin Disease Hospital, School of Medicine, Tongji University, Shanghai, China

**Keywords:** colon cancer, liver metastasis, database analysis, prognosis model, nomogram

## Abstract

**Objective:**

A proportional hazard model was applied to develop a large-scale prognostic model and nomogram incorporating clinicopathological characteristics, histological type, tumor differentiation grade, and tumor deposit count to provide clinicians and patients diagnosed with colon cancer liver metastases (CLM) a more comprehensive and practical outcome measure.

**Methods:**

Using the Transparent Reporting of multivariable prediction models for individual Prognosis or Diagnosis (TRIPOD) guidelines, this study identified 14,697 patients diagnosed with CLM from 1975 to 2017 in the Surveillance, Epidemiology, and End Results (SEER) 21 registry database. Patients were divided into a modeling group (n=9800), an internal validation group (n=4897) using computerized randomization. An independent external validation cohort (n=60) was obtained. Univariable and multivariate Cox analyses were performed to identify prognostic predictors for overall survival (OS). Subsequently, the nomogram was constructed, and the verification was undertaken by receiver operating curves (AUC) and calibration curves.

**Results:**

Histological type, tumor differentiation grade, and tumor deposit count were independent prognostic predictors for CLM. The nomogram consisted of age, sex, primary site, T category, N category, metastasis of bone, brain or lung, surgery, and chemotherapy. The model achieved excellent prediction power on both internal (mean AUC=0.811) and external validation (mean AUC=0.727), respectively, which were significantly higher than the American Joint Committee on Cancer (AJCC) TNM system.

**Conclusion:**

This study proposes a prognostic nomogram for predicting 1- and 2-year survival based on histopathological and population-based data of CLM patients developed using TRIPOD guidelines. Compared with the TNM stage, our nomogram has better consistency and calibration for predicting the OS of CLM patients.

## 1 Introduction

Globally, colon cancer is the third most common tumor and the second leading cause of cancer-related deaths (1, 2). The 5-year survival rate for colon cancer is 64.6%, while for synchronous metastasis, the patient’s survival is only 14.3%. Liver metastasis is the most frequently (17%) observed synchronous metastasis (3). It occurs in over 25% of patients initially and 50% of patients throughout the disease (4).

The prognosis of CLM varies significantly such that personalized prediction of CLM has become the focus of various studies, including those of the American Joint Committee on Cancer (AJCC) TNM system, which has been applied worldwide as the most authorized tool (5). However, the prediction accuracy of TNM staging is not satisfactory enough to predict outcomes (C-index=0.453) (6), which can relate to less predictors and classification on continuous variables (7).

Nomogram has been demonstrated to enhance predictive accuracy. Huang et al. developed two nomograms for the overall survival (OS) of patients with lung metastasis, having C-indexes of 0.754 and 0.749 (8). Furthermore, the C-index of a nomogram predicting the risk of bone metastasis has been reported to be 0.929 (9). Therefore, the present study will construct prognosis nomogram, providing clinicians with a more comprehensive outcome measure.

Recently, the identification of predictors was perceived great importance for prognosis nomogram. The histological type (10, 11), tumor differentiation grade (12), and tumor deposit count (13) have been recognized as independent predictors of colon cancer prognosis, as well as liver metastases. Several published studies have reported the prediction prognosis of histopathological predictors, respectively; however, insufficient sample sizes have limited the prognostic capabilities. Nevertheless, there have been a few large-scale CLM nomograms that have incorporated these histopathological indicators. The nomogram by Wu et al. ignored the influence of the histological type and the presence of synchronous metastasis and treatment information, which are important reasons for the nomogram’s low consistency (C-index=0.621).

Thus, based on data from the Surveillance, Epidemiology, and End Results (SEER) database, this study aims to develop a large-scale model and construct a nomogram that incorporates histological type, tumor differentiation grade, and tumor deposit count. TRIPOD guidelines were used for the development and verification process of the present study.

## 2 Methods

**Figure 1:**
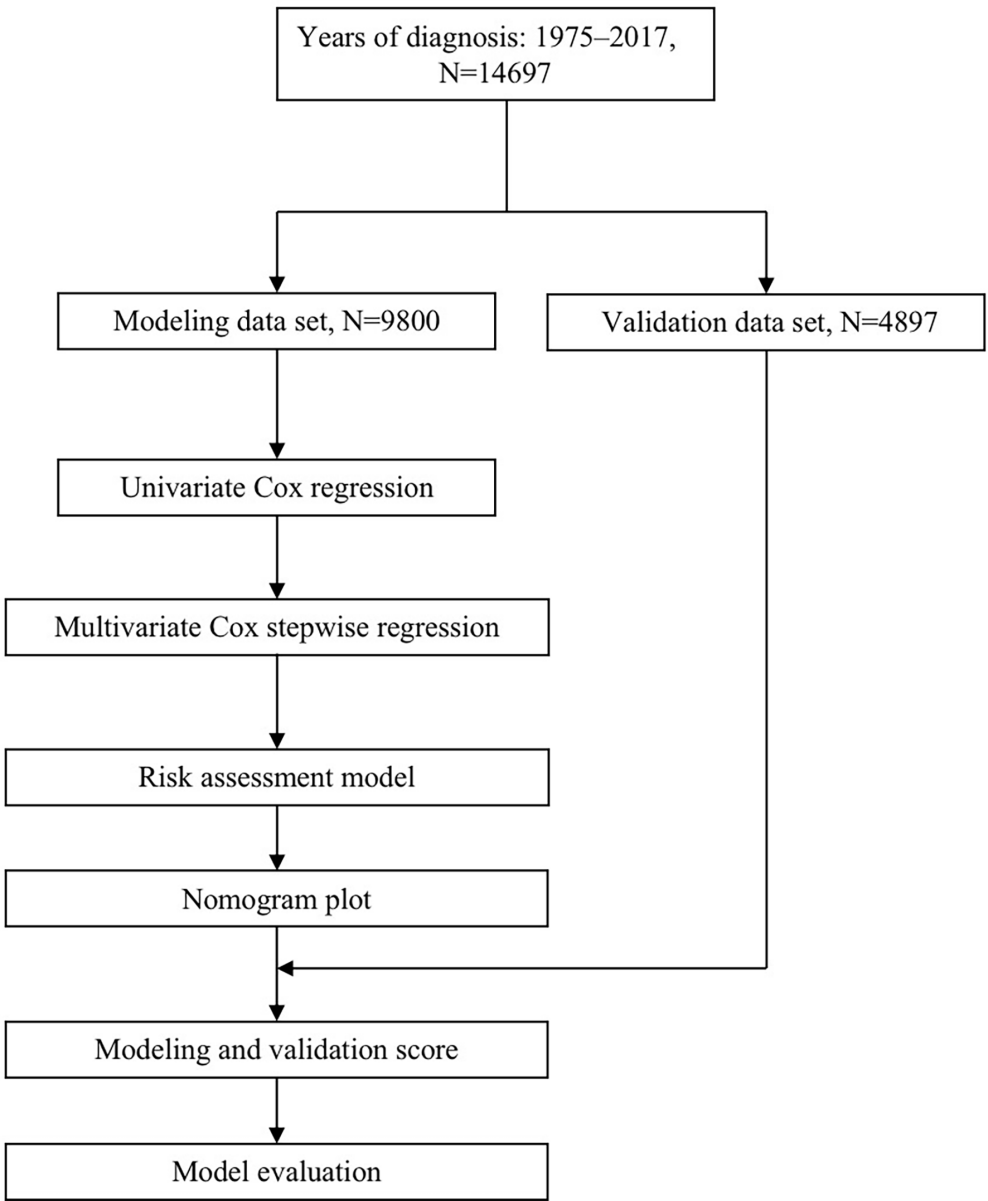
Flow diagram for predicting OS of CLM patients. OS, overall survival; CLM, colon cancer liver metastases.

This study design refers to the Prognosis Research Strategy (PROGRESS) (14). It uses the transparent reporting of a multivariable prediction model for individual prognosis or diagnosis (TRIPOD) to demonstrate its research plan (15). The study followed the TRIPOD checklist (**Supplementary Table 1**). The flow diagram is displayed in **Figure 1**.

### 2.1 Participants

The data for this study were extracted from information recorded in the SEER 21 registry (16) from 1975 to 2017. The SEER database is a public cancer information registration database supported by the National Cancer Institute, collecting data from population registries to provide information on survival.

The analyzed data in this study are obtained from the SEER database, which is a multi-institution and multi-population registry. The inclusion criteria are as follows: (1) diagnosed with colon cancers (Site recode of ICD-O-3/WHO 2008: C180–C189; https://seer.cancer.gov/siterecode/icdo3_dwhoheme/index.html); (2) liver metastases; (3) complete survival data and follow-up data; (4) basic demographic information, sex and age; (5) histological information, histological type (ICD-O-3 Hist/behave), tumor differentiation grade (17), and tumor deposit (CS site-specific factor 4) (18); (6) clinical information, such as AJCC TNM category, bone/brain/lung metastasis (SEER Combined Mets at DX-bone/brain/lung), tumor size (CS tumor size), tumor location (primary site) (19), lymph (CS lymph nodes) (20), and CEA (CS site-specific factor 1) (21); (7) therapeutic measures that whether received surgery or surgery (Surgery Primary Site) (22) or chemotherapy. Missing entries (more than 80% of item missing or over 15 items were unknown) were excluded from predictive modeling analyses. For each variable, subtypes with frequency less than 5% were merged, to reduce small sample bias.

### 2.2 Ethics

Because the SEER data were de-identified, the study did not require either institutional review board approval or the subject’s informed consent. All procedures are in compliance with the 1964 Declaration of Helsinki and its subsequent amendments and standards.

### 2.3 Outcome

The outcome of the present study was death overtime, which was already assessed in the database. The definition of the outcome indicators and measurement methods was the same for all patients. Data collection of outcome indicators was performed from population registries of the SEER database, whose proponents did not participate in statistical analysis.

### 2.4 Predictors

In this study, information on the patient’s clinicopathological factors was collected and included histopathological indicators, such as tumor differentiation grade, tumor deposit, and histological type; population-based indicators, including age and sex; tumor-related indicators, including tumor site, tumor size, lymph, CEA, and metastasis, as well as treatment-related indicators of chemotherapy and surgery (surgery in primary site). The main elements of the TNM category was also included. Predictors were reliable and straightforward for clinicians. Age, sex, surgical history, and chemotherapy history were obtained by the clinical inquiry; tumor-related indicators such as tumor size, grade, tumor site, synchronous metastasis, etc., were obtained through previous disease assessment, which is approachable for clinicians. The predictors were defined according to the SEER classification (16; https://seer.cancer.gov/).

Prediction models are often adapted to diverse data for an improved prediction (23), and subpopulations with small sample sizes had been merged or removed to increase the accuracy.

### 2.5 Missing Data

Missing data was marked as “unknown” and was estimated for the prognosis model, allowing patients who lack some data to use this model. However, missing entries (more than 80% of item missing or over 15 items were unknown) were excluded from predictive modeling analyses.

### 2.6 Statistical Analysis

#### 2.6.1 Modeling and Verifying Samples

A total of 14,757 patients were involved in this study. Patients were randomly divided into a modeling group (n=9800), internal verifying group (n=4897). An independent external verifying dataset (n=60) were obtained, which was completely independent from the data of model training, with varied demographic background characteristics. Clinical data were collected from medical record reviews in China.

#### 2.6.2 Cox Regression Modeling

Univariate regression analysis was used to determine predictors with independent effects and to initially screen predictors. In addition, multivariate stepwise Cox regression was used to analyze statistically significant variables by hazard ratios (HR) and to predict the association of overall survival and related prognostic factors.

#### 2.6.3 Nomogram Construction

A nomogram was constructed using R software after Cox regression analysis based on the modeling group. All statistical analyses were completed in R software (3rd edition 3.6.2; https://www.r-project.org).

### 2.7 Risk Groups

This study did not group the predicted probability into risk groups, as the prediction model is a basis for risk judgment.

### 2.8 Model Validation

Model internal verification was conducted using the SEER data sets, and the external validation was performed based on independent cohort. The consistency of the model was displayed using the C-index, the area under the receiver operating curves (AUCs). The model was acceptable when the AUC was over 0.7. The calibration curve was also used for model verification.

## 3 Results

### 3.1 Participants

**Figure 2:**
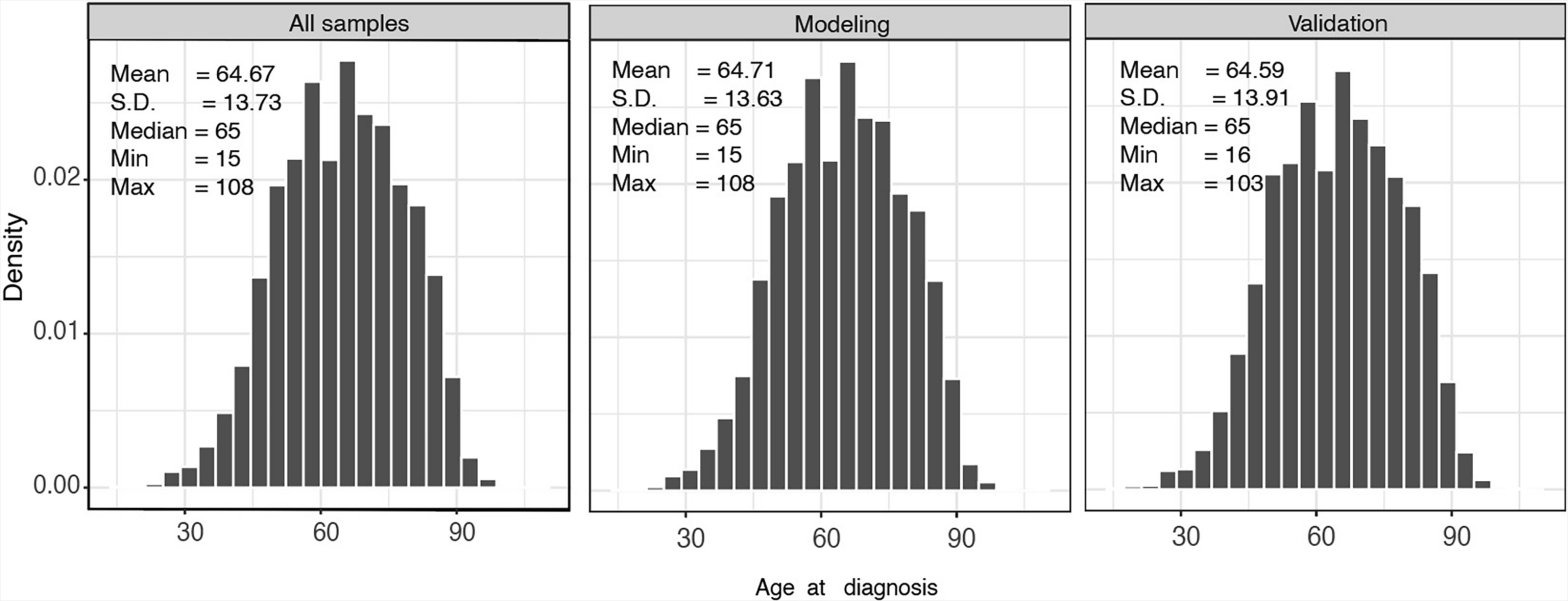
Histogram of the age distribution of all samples, modeling set, and validation set.

A total of 14,697 patients from SEER database were involved in this study. Patients were randomly divided into a modeling group (n=9800) and a verification group (n=4897). **Figure 2** shows the baseline characteristics of all patients stratified by age; the median age of the modeling group and validation group was 65 years. Patients with CLM are more likely to be male (54.38%), regardless of race or region.

Adenocarcinoma seems to be related to liver metastasis; l3,434 (91.41%) patients exhibited adenocarcinoma in the present study, while the remaining 1263 (8.59%) patients were divided into the group without adenocarcinoma for comparative analysis. Patients with a high degree of tumor differentiation grade are known to more likely metastasize; therefore, only 782 (5.32%) CLM patients presented as tumor differentiation grade I. In addition, our results demonstrated that tumor differentiation grade II (65.84%), or moderate differentiation, was present in the majority of CLM patients. Only 13.36% of patients presented positive in tumor deposit, which referred to a solitary tumor nodule that existed in the lymphatic drainage area of the primary tumor. Overall, 11,452 (77.92%) patients died, and the median survival was 14 months. Other population-based data are presented in **Table 1**.

**Table 1:** The baseline characteristics of enrolled CLM patients.

	All samples		Modeling		Validation		*P*
	N	Percentage (%)	N	Percentage (%)	N	Percentage (%)	
**Sex**							0.3268
Female	6705	45.62	4443	45.34	2262	46.19	
Male	7992	54.38	5357	54.66	2635	53.81	
**Tumor site**							0.7332
Colon, unknown	21	0.14	16	0.16	5	0.10	
Left colon	6135	41.74	4067	41.50	2068	42.23	
Overlapping lesion	234	1.59	154	1.57	80	1.63	
Right colon	7041	47.91	4706	48.02	2335	47.68	
Transverse colon	1266	8.61	857	8.74	409	8.35	
**Histological type**							0.0001
Adenocarcinoma	13434	91.41	9007	91.91	4427	90.40	
Not adenocarcinoma	1263	8.59	793	8.09	470	9.60	
**Tumor differentiation grade**							0.3698
II	9677	65.84	6470	66.02	3207	65.49	
IV	738	5.02	476	4.86	262	5.35	
I	782	5.32	507	5.17	275	5.62	
III	3500	23.81	2347	23.95	1153	23.55	
**T category**							0.6903
T0	3	0.02	2	0.02	1	0.02	
T1	1171	7.97	785	8.01	386	7.88	
T2	354	2.41	249	2.54	105	2.14	
T3	6055	41.20	4048	41.31	2007	40.98	
T4	4626	31.48	3077	31.40	1549	31.63	
TX	2488	16.93	1639	16.72	849	17.34	
**N category**							0.9058
N0	3872	26.35	2580	26.33	1292	26.38	
N1	5104	34.73	3480	35.51	1696	34.63	
N2	4740	32.25	3157	32.21	1583	32.33	
NX	981	6.67	655	6.68	326	6.66	
**Bone metastasis**							0.2317
No	14151	96.28	9423	96.15	4728	96.55	
Yes	546	3.72	377	3.85	169	3.45	
**Brain metastasis**							0.9976
No	14589	99.27	9728	99.27	4861	99.26	
Yes	108	0.73	72	0.73	36	0.74	
**Lung metastasis**							0.6191
No	12017	81.76	8002	81.65	4015	81.99	
Yes	2680	18.24	1798	18.35	882	18.01	
**Tumor deposit**							0.7094
No	6127	41.69	4068	41.51	2059	42.05	
Unknown	6607	44.95	4409	44.99	2198	44.88	
Yes	1963	13.36	1323	13.50	640	13.07	
**Tumor size**							0.7063
0-1cm	11664	79.36	7789	79.48	3875	79.13	
1-5cm	32	0.22	23	0.23	9	0.18	
Unknown	3001	20.42	1988	20.29	1013	20.69	
**Lymph**							0.5509
No	13445	91.48	8948	91.31	4497	91.83	
Unknown	1232	8.38	838	8.55	394	8.05	
Yes	20	0.14	14	0.14	6	0.12	
**CEA**							0.7122
Negative	1371	9.33	905	9.23	466	9.52	
Positive	8553	58.20	5725	58.42	2828	57.75	
Unknown	4773	32.48	3170	32.35	1603	32.73	
**Surgery**							0.8498
No	4274	29.08	2845	29.03	1429	29.18	
Yes	10423	70.92	6955	70.97	3468	70.82	
**Chemotherapy**							0.7205
No/Unknown	4980	33.88	3311	33.79	1669	34.08	
Yes	9717	66.12	6489	66.21	3228	65.92	

P-value was adopted on comparation of modeling and verifying dataset. CLM, colon cancer liver metastases; CEA, carcinoembryonic antigen.

### 3.2 Model Development

#### 3.2.1 Cox Regression to Screen Predictors

Univariate regression analysis was carried out to predict the overall survival of CLM patients. The findings showed that only tumor size of 1-5cm, lung metastasis, and tumor deposit counts had a significant association with higher incidence of death (**Table 2**). Meanwhile, chemotherapy and surgery were also identified as significant predictors. Notably, the interaction between factors would influence the results of univariate regression. Therefore, we carried out further multivariable regression. Stepwise regression was used for the multivariable analysis, and the results showed that all predictors were found to be significantly associated with OS, except for the T category and tumor site (**Table 3**).

**Table 2:** Univariate regression for OS.

	β	HR	se(β)	Z	*P*	95% CI (HR)	
**Age**	-0.003	0.997	0.002	-1.751	0.0799	0.994	1.000
**Sex**							
Female	Ref						
Male	0.015	1.015	0.043	0.355	0.7229	0.933	1.105
**Tumor site**							
Colon, unknown	Ref						
Left colon	-0.730	0.482	0.253	-2.883	0.0039	0.293	0.792
Overlapping lesion	-0.554	0.575	0.305	-1.818	0.0691	0.317	1.044
Right colon	-0.688	0.503	0.254	-2.708	0.0068	0.306	0.827
Transverse colon	-0.626	0.535	0.263	-2.381	0.0173	0.319	0.895
**Histological type**							
Adenocarcinoma	Ref						
Not adenocarcinoma	0.047	1.048	0.087	0.545	0.5858	0.885	1.242
**Tumor differentiation grade**							
II	Ref						
IV	-0.028	0.973	0.136	-0.203	0.8391	0.745	1.270
I	-0.001	0.999	0.084	-0.012	0.9906	0.848	1.178
III	-0.029	0.972	0.061	-0.473	0.6364	0.862	1.095
**T category**							
T0				Ref			
T1	1.448	4.256	1.006	1.440	0.1498	0.593	30.546
T2	1.421	4.140	1.006	1.413	0.1578	0.577	29.726
T3	1.267	3.550	1.002	1.265	0.2060	0.498	25.290
T4	1.245	3.474	1.002	1.242	0.2142	0.487	24.776
TX	1.628	5.091	1.004	1.621	0.1050	0.712	36.421
**N category**							
N0	Ref						
N1	-0.012	0.981	0.052	-0.376	0.7073	0.886	1.086
N2	-0.164	0.849	0.057	-2.858	0.0043	0.759	0.950
NX	0.061	1.063	0.129	0.474	0.6358	0.826	1.368
**Bone metastasis**							
No	Ref						
Yes	-0.089	0.915	0.187	-0.476	0.6340	0.634	1.320
**Brain metastasis**							
No	Ref						
Yes	0.871	2.390	0.449	1.942	0.0521	0.992	5.758
**Lung metastasis**							
No	Ref						
Yes	0.225	1.252	0.066	3.393	0.0007	1.100	1.426
**Tumor deposit**							
No	Ref						
Unknown	0.112	1.119	0.050	2.260	0.0238	1.015	1.233
Yes	0.386	1.472	0.061	6.307	0.0000	1.305	1.660
**Tumor size**							
0-1cm	Ref						
1-5 cm	1.064	2.897	0.501	2.123	0.0338	1.085	7.735
unknown	0.132	1.141	0.061	2.151	0.0315	1.012	1.286
**Lymph**							
No	Ref						
Unknown	0.143	1.153	0.104	1.376	0.1689	0.941	1.413
Yes	0.968	2.634	0.578	1.676	0.0938	0.849	8.176
**CEA**							
Negative	Ref						
Positive	-0.050	0.951	0.075	-0.669	0.5038	0.822	1.101
Unknown	0.025	1.025	0.079	0.313	0.7541	0.878	1.196
**Surgery**							
No	Ref						
Yes	-0.357	0.700	0.059	-6.052	0.0000	0.623	0.785
**Chemotherapy**							
No	Ref						
Yes	-0.025	0.976	0.061	-0.407	0.6841	0.867	1.099

OS, overall survival; HR, hazard ratio; CI, confidence interval.

**Table 3:** Multivariable regression for OS.

	β	HR	se(β)	Z	*P*
**Age**	0.014	1.010	0.001	15.150	<0.0001
**Sex**					
Female		Ref			
Male	0.082	1.090	0.023	3.540	0.0004
**Tumor site**					
Colon, unknown		Ref			
Left colon	31.34	5.05×10^5^	1.79×10^2^	0.073	0.9414
Overlapping lesion	34.22	6.74×10^5^	1.79×10^2^	0.075	0.9401
Right colon	33.83	6.48×10^5^	1.79×10^2^	0.075	0.9403
Transverse colon	33.99	6.58×10^5^	1.79×10^2^	0.075	0.9402
**Histological type**					
Adenocarcinoma		Ref			
Not adenocarcinoma	0.160	1.170	0.042	3.810	0.0001
**Tumor differentiation grade**					
II		Ref			
IV	0.544	1.720	0.052	10.510	<0.0001
I	-0.408	0.665	0.056	-7.230	<0.0001
III	0.385	1.470	0.027	14.080	<0.0001
**T category**					
T0		Ref			
T1	-0.399	0.671	1.000	-0.397	0.6916
T2	-0.925	0.397	1.010	-0.917	0.3589
T3	-0.556	0.573	1.010	-0.554	0.5800
T4	-0.281	0.755	1.010	-0.280	0.7794
TX	-0.383	0.682	1.000	-0.382	0.7026
**N category**					
N0		Ref			
N1	0.151	1.160	0.033	4.618	<0.0001
N2	0.455	1.580	0.037	12.460	<0.0001
NX	0.166	1.180	0.051	3.281	0.0010
**Bone metastasis**					
No		Ref			
Yes	0.362	1.440	0.056	6.426	<0.0001
**Brain metastasis**					
No		Ref			
Yes	0.432	1.540	0.125	3.460	<0.0001
**Lung metastasis**					
No		Ref			
Yes	0.278	1.320	0.030	9.285	<0.0001
**Tumor deposit**					
No		Ref			
Unknown	0.197	1.220	0.032	6.250	<0.0001
Yes	0.112	1.120	0.038	2.943	0.0032
**Tumor size**					
0-1cm		Ref			
1-5cm	0.296	1.340	0.231	1.281	0.2003
unknown	-0.082	0.921	0.037	-2.255	0.0241
**Surgery**					
No		Ref			
Yes	-0.806	0.447	0.049	-16.462	<0.0001
**Chemotherapy**					
No		Ref			
Yes	-1.086	0.338	0.026	-42.532	<0.0001

OS, overall survival; HR, hazard ratio; CI, confidence interval.

Based on these results, the Cox modeling in this study included twelve independent predictors (age, sex, histological type, tumor differentiation grade, tumor deposit, N category, bone metastasis, brain metastasis, lung metastasis, tumor size, surgery, and chemotherapy) of OS. As an essential indicator of the TNM system, the T category was included in the construction of the model. The predictive performance indicates that significant unknown factors remain (coded as 999, unknown or NOS in SEER database) for objective reasons, including data cannot be assessed, therefore, they may be of particular clinical significance (24).

#### 3.2.2 Prognosis Model Development

Taking 0.05 as threshold of *P*-value, we used the hazard ratio (HR) values and coefficient of the multivariable regression model to represent the parameters of the independent variables for death. We identified a predictor as a risk factor for death when the corresponding coefficient was>0, or the HR value was>1 significantly. Among these clinical features, tumor differentiation grade IV was the most important factor for prognosis (β=0.544, HR=1.720), which indicated that the risk of death in grade IV was 1.720 times that of grade II followed by tumor differentiation grade III (β=0.385, HR=1.470). Conversely, tumor differentiation grade I (β=-0.408, HR=0.665) was considered a protective factor for CLM OS. In different histological types, not exhibiting adenocarcinoma (β=0.160, HR=1.170) resulted in a poorer prognosis, the risk of which was 1.17 times that of adenocarcinoma patients. In addition, the occurrence of tumor deposit counts (β=0.112, HR=1.120) also increased the risk of death by 1.12 times.

As for synchronous metastasis, the prognosis of patients with brain metastasis (β=0.466, HR=1.540) was worse, and the impact on prognosis was more incredible than bone metastasis (β=0.362, HR=1.440) or lung metastasis (β=0.278, HR=1.320). Although the influence of sex is not apparent, it can be concluded from the model that the risk of death for men was 1.09 times that of women. The risk of death for patients who received surgery (β=-0.806, HR=0.447) was only 45% of those who did not, and the chemotherapy seems to be more effective in improving OS (β=-1.086, HR=0.338).

#### 3.2.3 Nomogram Construction

**Figure 3:**
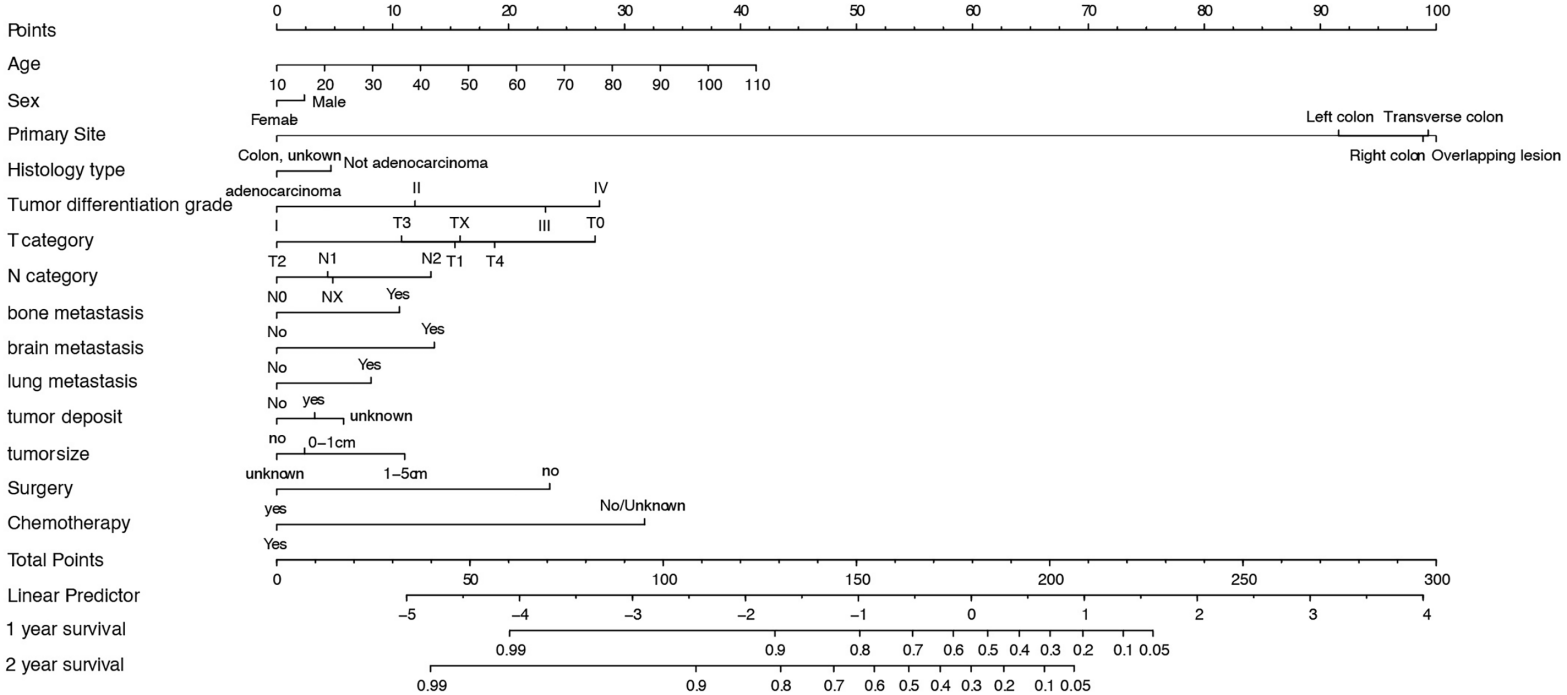
Nomogram for predicting 1- and 2-year OS of CLM patients. SD, Standard Deviation; OS, overall survival; CLM, colon cancer liver metastases.

Based on the multivariable regression model results, specific scores of each predictor and total scores were plotted (**Figure 3**). The score predictors were as follows: male, 2.5; not adenocarcinoma, 5; tumor differentiation grade II, 12.5; tumor differentiation grade III, 22.5; IV, 27.5; tumor deposit, 2.5; bone metastasis, 10; brain metastasis, 12.5; lung metastasis, 7.5; no surgery, 25; and no chemotherapy, 30. Applying this model for clinical prognosis prediction is quite convenient. Consider a female patient aged 70 years old, having a 4-cm-long tumor in the left colon, tumor differentiation grade II, and histological type of adenocarcinoma, without tumor deposit; grade T2, N1, and lung metastasis, who received chemotherapy, but no surgery. Her total score is 174, and clinicians could draw a vertical line at the corresponding position to obtain the corresponding predicted survival rate. Clinicians can thus use the visual nomogram to directly calculate the scores of the indicators and get the survival rate of patients to formulate suitable individual treatments.

### 3.3 Model Validation

**Figure 4:**
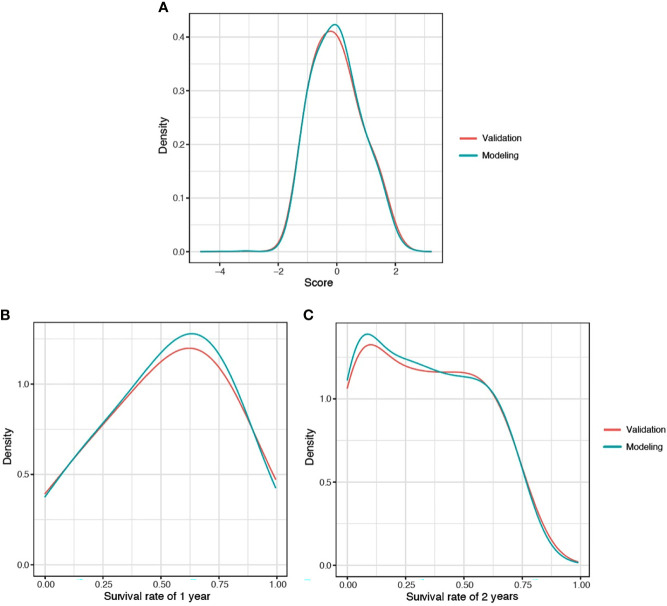
Distribution plot for score **(A)**, 1-year **(B)**, and 2-year **(C)** survival rate of CLM patients. CLM, colon cancer liver metastases.

**Figure 5:**
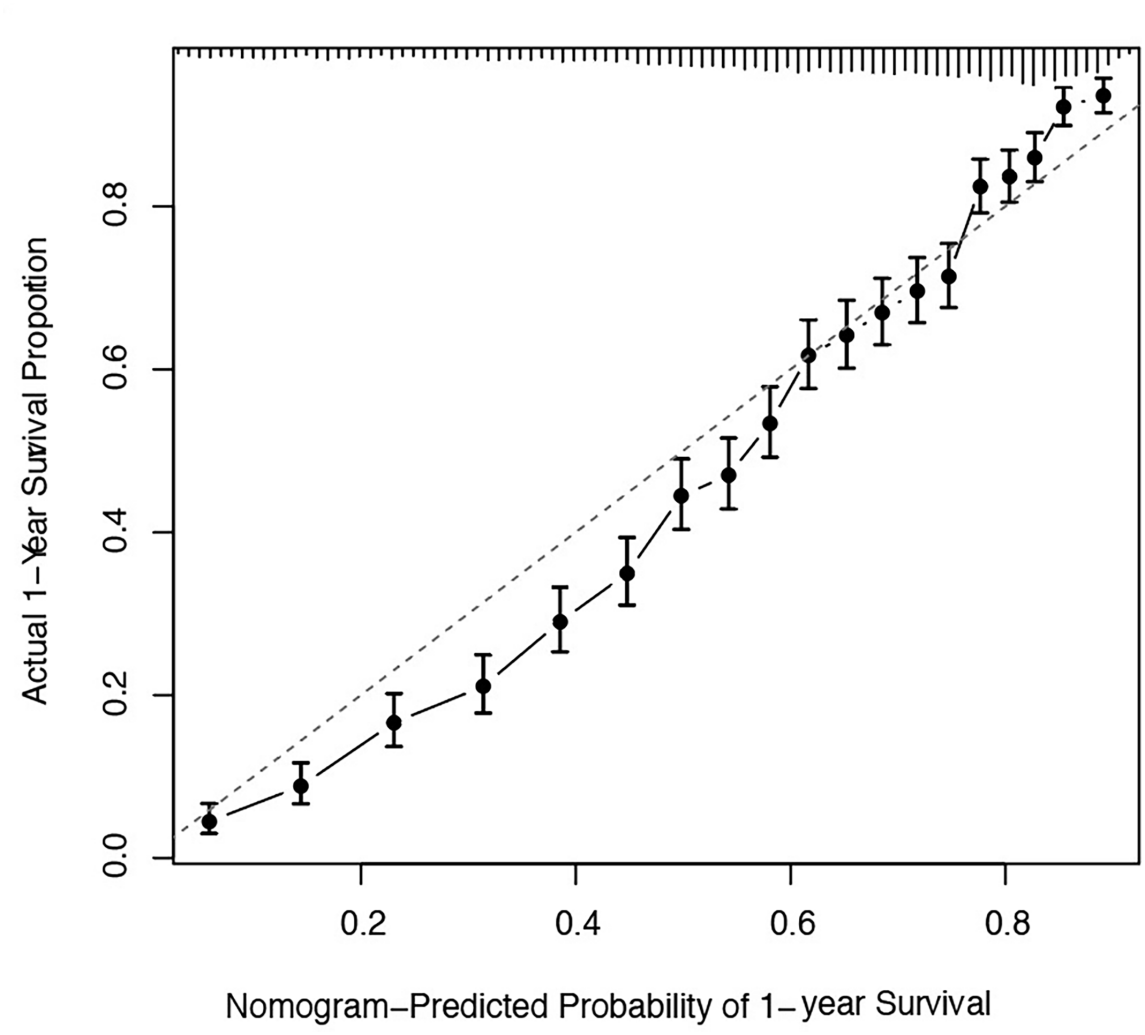
Calibration plots of the nomogram for 1-year and 2-year prediction for OS of CLM patients. OS, overall survival; CLM, colon cancer liver metastases.

**Figure 6:**
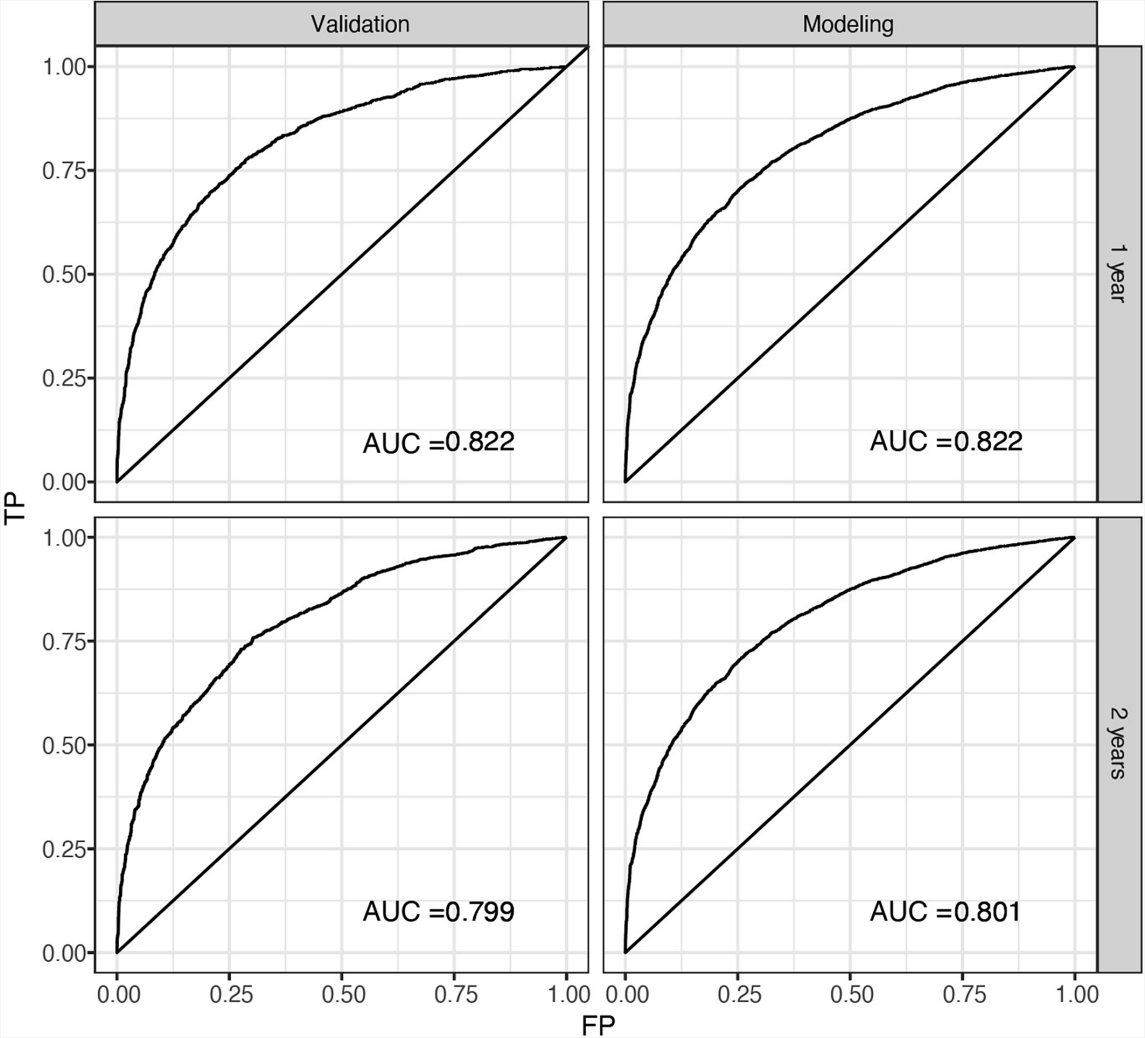
ROC of the nomogram for 1-year and 2-year internal prediction. ROC, receiver operating curves, AUC, area under the curve; FP, false positive; TP, true positive.

**Figure 7:**
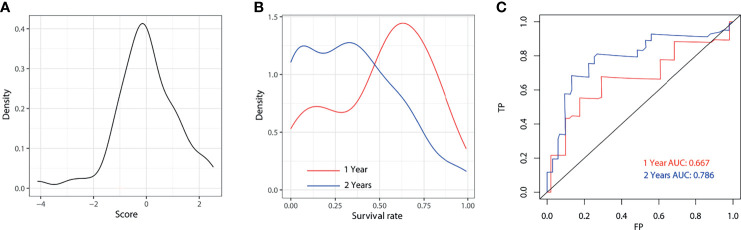
External validation for 1-year and 2-year of the model. Distribution plot for score **(A)**, 1-year and 2-year survival rate **(B)**. ROC of the nomogram for 1-year and 2-year external validation **(C)**. AUC, area under the curve; FP, false positive; TP, true positive.

The predicted distributions of the score, 1-year survival, and 2-year survival in the modeling and internal validation groups were consistent (**Figure 4**). A calibration plot of the 1-year OS demonstrated good calibration between prediction and actual survival (**Figure 5**). The C-indexes of the modeling group and internal verification group were 0.751 and 0.752 (**Table 4**). The ROC curve analysis (**Figures 6**, **7**) was used for nomogram validation, and the value of the area under the curve (AUC) represented the consistency of the model. The results showed that the AUC of the modeling group was 0.822 at 1-year and 0.801 at 2-year, and in the internal verification group, the AUC was 0.822 for 1-year and 0.799 for the 2-year survival (mean AUC=0.811). The ROC analysis of external verification (**Figure 7** and **Supplementary Table 2**) indicated a good predictive performance of the model, in which the AUC values for predicting 1- and 2-year OS were 0.786 and 0.667 respectively. Albeit on small samples, this nomogram showed reliable predictive power in the external discrimination test (mean AUC=0.727).

**Table 4:** Multivariable regression for OS.

Data set	C	Dxy	aDxy	SD	Z	P	n
Modeling	0.751	-0.502	0.502	0.006	89.56	0	9800
Validation	0.752	-0.498	0.498	0.008	62.41	0	4897

## 4 Discussion

In this study, we constructed a predictive model for CLM patients using multi-institutional clinical data from the SEER database. The prognosis nomogram for OS was established and its consistency and calibration were verified.

The predictors included in the prognostic nomogram were age, sex, tumor site, histological type, tumor differentiation grade, T category, N category, bone metastasis, lung metastasis, brain metastasis, tumor deposit, surgery, and chemotherapy.

Multivariate regression analysis indicated that age was an essential prognostic risk factor. Several models have shown that age is a risk factor (25, 26), illustrating that the elderly have a poorer prognosis, especially when over 60 years old (6). Our nomogram demonstrated that tumor differentiation grade IV, undifferentiated, has a poorer prognosis than tumor differentiation grade I, well-differentiated. Consistent with this result, the tumor differentiation grade was observed to be associated with the faster growth rate and more aggressive ability of tumor cells, leading to distant metastasis of CLM (27) and OS. Tumor deposits were found to be a statistically significant factor, which may be considered as a third mixed pathway of tumor migration and invasion in perivascular, perineural, or mesentery (28).

What differed from previous studies was that T0 and T1 were identified as risk factors for CLM, which may be related to the highly malignant characteristics of the tumors in the early stages (9). Adenocarcinomas, such as mucinous adenocarcinoma or signet ring cell carcinoma, are more likely to undergo peritoneal metastasis (29), always leading to poorer prognosis. Still, early mucinous adenocarcinoma may have a better prognosis. However, our results indicate that patients with adenocarcinoma will have a better prognosis, which may be related to the classification of the histological subtypes, but this needs further research to be validated.

Validation of the nomogram resulted in a reliable ability to discriminate events. The AUC has been generally accepted and widely used for model validation. The model achieved excellent prediction power on both internal (mean AUC=0.811) and external validation (mean AUC=0.727), respectively, which were substantially superior to the American Joint Committee on Cancer (AJCC) TNM system. This study will provide clinicians and patients diagnosed with colon cancer liver metastases (CLM) a more comprehensive and practical outcome measure, to help clinicians assess patient prognosis and determine personalized treatment decisions.

This study developed and validated a prognosis model to predict the survival of CLM patients under the guidance of both the PROGRESS framework and the TRIPOD prediction research report for the first time. Previously developed nomograms (30–33) could be more convincing if they expanded their sample size. We included as much population as possible from the SEER database, covering 35% population of the US. Several predictive models of CLM had been published to predict the survival rate of patients after hepatectomy (34, 35), while patients who were suitable for hepatectomy accounted for only 6.1% of patients (36), which led to the limitation of the model in clinical use. Wu Q et al. developed nomograms of CLM from the SEER database but excluded histological type, and synchronous metastasis, without any treatment-related data. This study incorporated these significant predictors, including more available clinical indicators as predictors to improve model applicability.

Internal and external validation of the current nomogram demonstrated high accuracy in predicting OS of CLM patients. However, three limitations must be resolved. The first problem relates to the inevitable lack of other clinicopathological factors of the SEER data set, some vital prognostic factors of liver metastasis, such as the type of liver resection, should be included in future research. Secondly, model focusing on subtypes of adenocarcinoma, or primary surgery type should be performed furtherer, using specific data set. Furthermore, although the external verification indicated an excellent predictive effect, the AUC value of 1-year OS prediction was slightly lower than 2-year, leading requirement to better external validation using large samples of independent multicenter cohorts.

## 5 Conclusion

In conclusion, based on the TRIPOD prediction research report, we established and validated nomograms to predict the 1- and 2-year survival based on histopathological and population-based data of CLM patients. Compared with the traditional staging system TNM, our nomogram achieved relatively good discrimination and calibration.

## Data Availability Statement

The original contributions presented in the study are included in the article/**Supplementary Material**. Further inquiries can be directed to the corresponding authors.

## Author Contributions

LK and YZ conceived and designed the study and provided administrative support. YL, WL, and X-DL collected and assembled the data. H-PZ and T-YL performed the external validation. S-YY and BL analyzed and interpreted the data. All authors contributed to the article and approved the submitted version.

## Funding

This study was supported by the National Key Research and Development Program of China (No. 2018YFC1705305), the NSFC of China (No. 81973860, 81904214, 82004235), the Shanghai Development Office of TCM [No. ZY(2018-2020)-FWTX-4010, ZY(2018-2020)-FWTX-1008], and Dermatology Department of Traditional Chinese Medicine, Clinical Key Specialty Construction Project of Shanghai (No. shslczdzk05001), Xinglin Youth Scholar of Shanghai University of Traditional Chinese Medicine (no. RY411.33.10), Shanghai Sailing Program (no. 21YF1448100), and Peiran Scholar of Shanghai University of Traditional Chinese Medicine (no. 2015811038).

## Conflict of Interest

Authors H-PZ and T-YL were employed by Research and Development Center, Shanghai Applied Protein Technology Co., Ltd.

The remaining authors declare that the research was conducted in the absence of any commercial or financial relationships that could be construed as a potential conflict of interest.

## Publisher’s Note

All claims expressed in this article are solely those of the authors and do not necessarily represent those of their affiliated organizations, or those of the publisher, the editors and the reviewers. Any product that may be evaluated in this article, or claim that may be made by its manufacturer, is not guaranteed or endorsed by the publisher.
